# Breast Cancer knowledge, perceptions and practices in a rural Community in Coastal Kenya

**DOI:** 10.1186/s12889-019-6464-3

**Published:** 2019-02-12

**Authors:** Shahin Sayed, Anthony K. Ngugi, Megan R. Mahoney, Jaameeta Kurji, Zohray M. Talib, Sarah B. Macfarlane, Theresa A. Wynn, Mansoor Saleh, Amyn Lakhani, Esther Nderitu, Felix Agoi, Zul Premji, Jo Anne Zujewski, Zahir Moloo

**Affiliations:** 1 Department of Pathology, Faculty of Health Sciences - East Africa, Aga, Khan University Nairobi, 3rd Parklands Avenue, P.O. Box 30270-00100, Nairobi, Kenya; 2 Centre for Population, Faculty of Health Sciences - East Africa, Aga Khan University, Nairobi, 3rd Parklands Avenue, P.O. Box 30270-00100, Nairobi, Kenya; 3 Department of Medicine General Medicines Discipline Stanford, University, Palo Alto-C, 291 Campus Dr.Palo Alto, California, CA 94305 USA; 4 University of Ottawa, School of Epidemiology, Public Health & Preventive Medicine, Alta Vista Campus 600 Peter Morand Crescent,Ottawa, Ontario, K1G 5Z3 Canada; 5 Department of Medicine and of Health Policy and Management, The George Washington University (GWU) Medical School, Ross Hall, 2300 Eye Street, Washington D.C., NW 20037 USA; 6 Department of Epidemiology and Biostatistics, School of Medicine, University of California San Francisco, San Francisco, Mission Hall: Global Health & Clinical Sciences Building 550 16th Street, 2nd Floor Box #0560, San Francisco, CA 94158-2549 USA; 7 University of Alabama at Birmingham, School of Medicine, Division of Preventive Medicine, University of Alabama, Medical Towers, MT-621. 1720 2nd Ave South, Birmingham, AL 35294-4410 USA; 8 Department of Medicine, University of Alabama Comprehensive Cancer Center, Birmingham, Alabama, USA Wallace Tumor Institute, WTI 202. 1720 2nd Ave South, Birmingham, AL 35294-3300 USA; 9 Mombasa Research Office, Faculty of Health Sciences - East Africa, Aga Khan University, 3rd Parklands Avenue, P.O. Box 30270-00100, Nairobi, Kenya; 10 School of Nursing and Midwifery, Aga Khan University Nairobi Kenya, 3rd Parklands Avenue, P.O. Box 30270-00100, Nairobi, Kenya; 11 Mombasa Research Office, Faculty of Health Sciences - East Africa, Aga Khan University, 3rd Parklands Avenue, P.O. Box 30270-00100, Nairobi, Kenya; 12 JZ Oncology, JZ Oncology , 4525 North Chelsea Lane, Bethesda, MD 20814 USA

**Keywords:** Breast health care, Breast cancer, Knowledge, Attitudes, Barriers, Male heads of households, Kenya

## Abstract

**Background:**

Data on breast healthcare knowledge, perceptions and practice among women in rural Kenya is limited. Furthermore, the role of the male head of household in influencing a woman’s breast health seeking behavior is also not known. The aim of this study was to assess the knowledge, perceptions and practice of breast cancer among women, male heads of households, opinion leaders and healthcare providers within a rural community in Kenya. Our secondary objective was to explore the role of male heads of households in influencing a woman’s breast health seeking behavior.

**Methods:**

This was a mixed method cross-sectional study, conducted between Sept 1st 2015 Sept 30th 2016. We administered surveys to women and male heads of households. Outcomes of interest were analysed in Stata ver 13 and tabulated against gender. We conducted six focus group discussions (FGDs) and 22 key informant interviews (KIIs) with opinion leaders and health care providers, respectively. Elements of the Rapid Assessment Process (RAP) were used to guide analysis of the FGDs and the KIIs.

**Results:**

A total of 442 women and 237 male heads of households participated in the survey. Although more than 80% of respondents had heard of breast cancer, fewer than 10% of women and male heads of households had knowledge of 2 or more of its risk factors.

More than 85% of both men and women perceived breast cancer as a very serious illness. Over 90% of respondents would visit a health facility for a breast lump.

Variable recognition of signs of breast cancer, limited decision- autonomy for women, a preference for traditional healers, lack of trust in the health care system, inadequate access to services, limited early-detection services were the six themes that emerged from the FGDs and the KIIs. There were discrepancies between the qualitative and quantitative data for the perceived role of the male head of household as a barrier to seeking breast health care.

**Conclusions:**

Determining level of breast cancer knowledge, the characteristics of breast health seeking behavior and the perceived barriers to accessing breast health are the first steps in establishing locally relevant intervention programs.

**Electronic supplementary material:**

The online version of this article (10.1186/s12889-019-6464-3) contains supplementary material, which is available to authorized users.

## Background

Breast cancer accounts for 23% of all cases of female cancer in Kenya [1]. The majority of patients present with late stage and locally advanced disease, resulting in high mortality rates [2]. A necessary first step to improve breast cancer outcomes is improving breast healthcare and breast cancer knowledge in local communities. Another necessary step to mitigate late presentation of the disease is understanding the local barriers to early detection, accurate diagnosis and appropriate treatment [3].

The limited data for breast cancer incidence that are available from Kenya are predominantly urban, [1] and hospital-based [4]. It is likely that breast cancer is also a major health problem in the underserved rural areas of Kenya, but currently very little data exist on breast cancer incidence, breast cancer knowledge and health seeking practice of women from these populations. Women in low- and middle-income countries (LMICs) like Kenya may face multiple socio-economic, religious, cultural, health care provider and health systems barriers to accessing optimal breast cancer care [5].

In many rural communities of Kenya, men are important decision-makers in the family, however, there are no studies evaluating the influence of men in a woman’s breast healthcare seeking behaviour. Furthermore, there are no studies from Kenya directly assessing the knowledge of breast cancer among men.

We conducted a mixed methods study within a rural coastal community of Kenya to inform effective design of future interventions to improve access to breast cancer services. The aim of this study was to assess knowledge, perceptions and practices related to breast cancer among women, male heads of households, opinion leaders and healthcare providers in the coastal community of Kaloleni sub-county, Kilifi County, Kenya. Our secondary objective was to explore the role of male heads of households in influencing a woman’s breast health seeking behavior.

## Methods

### Study area and period

This study was performed in Kaloleni, a sub-county of Kilifi County, Kenya, between Sept 1st 2015 and Sept 30th 2016.

### Study design and population

The convergence model of a triangulation design was used in this mixed methods study (6).

In this one-phase approach, quantitative and qualitative data were collected and analyzed separately and assigned equal importance, and the results of each component were then compared to achieve a broader understanding of the phenomenon from a diversity of perspectives [6]. The quantitative component comprised female member and male head of household surveys. The qualitative part consisted of focus group discussions (FGDs), involving opinion leaders, and key informant interviews (KIIs) of health care providers. According to the 2009 Kenya National Population and Housing Census, Kaloleni had a population of about 290,000 living in about 44,000 households, with women of reproductive age comprising 25% of the population [7]. Approximately 70% of the population lives below the poverty line and 81% of residents rely on subsistence agriculture for their livelihood [8]. The Aga Khan University - East Africa (AKU-EA) and the University of California, San Francisco (UCSF), in partnership with the Ministry of Health of Kaloleni sub-county, Kilifi County, Kenya, run an Integrated Primary Health Care (IPHC) programme in Kaloleni sub-county. The IPHC programme has developed and maintains a regularly updated population registry of two Community Units (CUs), which provides a sampling frame of 2700 households and 16,005 residents and up-to-date denominator data for AKU - East Africa population-based studies.

The two study site CUs are served by one dispensary (the Kinarani Dispensary) and 100 Community Health Workers (CHWs) who are volunteers selected by the community to implement the community health strategy at the primary level. The Kinarani Dispensary and other nearby dispensaries provide basic primary care and these are linked to the sub-county (district) hospital at Mariakani Township. The Mariakani sub-county hospital is staffed by nurses, clinical officers and 8 medical officers who provide care across all health disciplines. All complex cases (including mastectomies) are referred to the tertiary level Coast Provincial General Hospital in the neighboring Mombasa County.

### Sample size calculation and sampling techniques

We randomly selected women aged 15–49 years from the IPHC database. Male heads of households were also selected at random from the same households where women were interviewed. This was a simple random selection without replacement. We did not set an age limit when selecting the males. For the quantitative part of the study, the sample size was based on the formula (Z_1-α/2_)^2^*(p(1-p))/d^2^, where (Z_1-α/2_) = 1.96, p = the proportion with knowledge of screening methods, which is 40% among women in this region (unpublished pilot data), and d = 0.05, which was the knowledge variable with the lowest frequency in this population . We added 15% to the calculated sample size, to compensate for anticipated non-response, bringing the total sample size to 425 women. For our secondary objective, a total of 237 male heads of households were sampled randomly from 50% of the households from which female respondents were sampled (and this figure also included an additional 10% to compensate for anticipated non-response). For the qualitative part of the study, specific groups were targeted for the FGDs and KIIs based on demographics, societal rank, or occupation. The responsibility for selection of the participants for the FGDs and the KIIs was vested with the local Ministry of Health officials.

### Data collection tools and procedures

Prior to the fieldwork, the study team developed survey tools for males, females, the focus group discussions and the key informant interviews (Additional files 1, 2, 3, 4). The male survey tool had additional questions specifically addressing their attitude towards the health seeking behavior of the women in the household. The male and female survey tools were reviewed, translated into Kiswahili and the local Giriama language, and independently back-translated into English. There were a total of 55 questions in each of these tools, which included the following domains: socio-demographic characteristics; knowledge of breast cancer risk factors, symptoms and signs; knowledge of screening and diagnosis methods; and the perceived barriers to accessing breast health care. The FGD tool was adapted from the Racial and Ethnic Approaches to Community Health (REACH) 2010 Program of the Division of Preventive Medicine, Department of Medicine, University of Alabama at Birmingham. Some questions were revised to be appropriate in the local context. The KII tool was adapted from the Central Texas Affiliate of Susan G. Komen® (Qualitative Data: Ensuring Community Input). Ten field enumerators (CHWs), were trained on breast healthcare and the study protocol and tools by the study team. We piloted the study tools in a neighboring village. Based on the feedback provided by the CHWs, the questionnaires were further refined and adopted.

### Focus group discussions

The study team conducted FGDs in Kiswahili and the local dialect at the Kinarani Dispensary. Facilitators were divided into 2 groups, each responsible for conducting three FGDs. A total of 6 groups participated; religious leaders, women elders, traditional healers, women’s group, an administration group comprising chiefs and assistant chiefs, and a group made up of the youth). Inclusion criteria for each FGD were distinct from each other and based on occupation/profession, thus maintaining participant homogeneity. No participant was enrolled in more than one FGD.

All FGDs were recorded. The field coordinator (FA) moderated the first session to set the pace and demonstrate the use of non-technical language, making it easy for the participants to understand terminologies. Each FGD session lasted about an hour and consisted of 17 questions to gain insight into participant understanding of cancer in general and breast cancer specifically, knowledge of screening and treatment options, and experiences in accessing health care services.

### Key informant interviews

The study investigators (ZM, SS, and EN) and field coordinator (FA) conducted Key Informant Interviews in English and Kiswahili, as appropriate. The one-on-one KIIs targeted medical and clinical officers, nurses and technologists within Kaloleni. A total of 25 questions were administered, interrogating the knowledge and perceptions of the health care providers on breast cancer symptoms and signs, screening activities, challenges faced in provision of breast health care services, and perceived barriers that may result in delays in diagnosis. The KIIs also lasted about an hour and were audio recorded.

### Data quality control

The interviewers (CHWS) administered the survey tools two weeks after their onsite training. Pairs of interviewers conducted the female and male interviews separately, but during the same household visit. The field coordinator (FA) undertook random onsite quality checks. Trained data clerks double-entered the data, which were verified in Epi Info v7.

### Data analysis

Quantitative survey data analysis was performed in Stata ver13. The distributions of the knowledge, perceptions and practice results were tabulated, overall by gender (Additional files 5, 6). Coding and analysis of the qualitative data was modeled after the Rapid Assessment Process [9], specifically (a) a multidisciplinary group was engaged to apply codes; and (b) an iterative process within the team was used to develop the coding system. Five researchers (MM, ZT, SS, TW and JK) each independently coded the first five transcripts, to establish inter-rater reliability, and then the rest of the transcripts were distributed across these five researchers for coding. New codes were added throughout the analysis. Themes were identified when coding saturation and thematic saturation was achieved (9).

## Results

### Quantitative findings

The interviewers administered 442 female and 237 male questionnaires. The median (IQR) ages of the study population were 22 (19–25) years for women and 31 (24–43) years for men. Almost equal proportions of women (70%) and men (73%) were married. Forty-eight percent (48%) of women had no formal education, and another 42% attended only primary school; the corresponding figures for men were 20 and 56%, respectively (Table 1).

**Table 1:** Socio-demographic characteristics of female members and male heads of households in Kaloleni sub-county, Kenya

	Females (*n* = 442)	Males (*n* = 237)
Median age (IQR) years	22 (19–25)	31 (23–43)
Characteristic	n(%)^a^	n(%)^a^
Marital status		
Married	307(70.7)	173(73.6)
Single	70(16.1)	49(20.9)
Widowed	40(9.1)	8(3.4)
Divorced	17(3.9)	5(2.1)
Highest level education		
None	212(48.5)	48(20.3)
Primary	182(41.6)	133(56.4)
Secondary	30(6.9)	44(18.6)
Tertiary	8(1.8)	9(3.8)
Adult education	5(1.1)	2(0.8)
Religion		
Protestant	185(42.1)	63(26.8)
Muslim	70(15.9)	67(28.5)
Catholic	34(7.7)	48(20.4)
Traditional	56(12.8)	16(6.8)
Other/None^b^	94(21.4)	41(17.4)
Occupation		
Farmer	136(31.1)	90(38.1)
Trader	47(10.7)	36(15.3)
Crafts	1(0.2)	25(10.6)
Technical	3(0.7)	9(3.8)
Clerk/management/administration	8(1.8)	3(1.3)
Housewife	77(17.6)	NA
Other^c^	64(14.6)	43(18.2)
None	102(23.3)	30(12.7)

*IQR* Inter-quartile range; *NA* Not applicable

^a^ total numbers may not add up to 442 (females) or 237 (males) because some respondents did not respond to specific questions

^b^Females, Other (*n* = 1), None (*n* = 93); Males, Other (n-1), None (*n* = 40)

^c^several (> 50 work categories mentioned)

Eighty-five percent of women and 96% of men had heard of cancer, and 82% of women and 92% of men had heard of breast cancer (Table 2). Thirty-five percent of women and 45% of men knew someone personally who had had breast cancer. The great majority of women and men (> 85% each) perceived breast cancer to be a very serious illness. Forty-five percent of women and 27% of men said they knew “nothing at all” about breast cancer.

**Table 2:** Knowledge and perceptions of breast cancer among female members and male heads of households in Kaloleni sub-county, Kenya

	Females (*n* = 442)	Males (*n* = 237)
Question	n(%)^a^	n(%)^a^
Have you heard of cancer?		
Yes	374(85.4)	227(96.2)
No	64(14.6)	9(3.8)
Have you heard of breast cancer?		
Yes	357(81.5)	217(92.3)
No	81(18.5)	18(7.7)
Have you known someone with BC?		
Yes	151(34.6)	105(44.5)
No	286(65.5)	131(55.5)
How much do you know about BC?		
Nothing at all	193(44.8)	64(27.2)
Only heard the term	88(20.4)	40(17.0)
A little	134(31.1)	122(51.9)
Very familiar	14(3.3)	9(3.8)
Is BC a serious illness?		
Yes	226(85.6)	162(94.2)
No	3(1.1)	1(0.6)
Don’t know	35(13.3)	9(5.2)
What causes BC?		
Don’t know	407(93.6)	216(92.3)
Other^b^	24(5.52)	18(7.7)
Do men get BC?		
Yes	43(9.9)	76(33.8)
No	159(36.6)	56(29.9)
Don’t’ know	233(53.6)	93(41.3)
Is BC curable?		
Yes	210(48.0)	142(60.4)
No	68(15.5)	33(14.0)
Don’t know	160(36.5)	60(25.5)
Can you survive BC if it is detected early?		
Yes	255(58.2)	171(72.8)
No	47(10.7)	26(11.1)
Don’t know	136(31.1)	38(16.2)
Can a traditional healer treat BC?		
Yes	17(3.9)	7(3.0)
No	334(77.1)	188(80.3)
Don’t know	82(18.9)	39(16.7)

*BC* = breast cancer

^a^ total numbers may not add up to 442 (females) or 237 (males) because some respondents did not respond to specific questions

^b^Including viruses, close contact with a person with BC, heredity, lifestyle, evil eye and witchcraft

Only 27% of the women and 40% of the men were aware of two or more major signs of breast cancer (Table 3). Similarly, knowledge of breast cancer early detection methods was low, with only 29% of women and 45% of men indicating that they had heard of mammography, clinical breast examination or breast self-examination. Among the reasons cited by women for not practicing breast self-examination, the most common were lack of knowledge about BSE (45%) and the perception that they had no apparent breast problems (39%). Only 6% had ever had a clinical breast exam (Table 3).

**Table 3:** Knowledge of breast cancer risk factors, signs, symptoms and diagnostic and screening methods among female members and male heads of households in Kaloleni sub-county, Kenya

	Females (n = 442)	Males (*n* = 236)
Question	n(%)^c^	n(%)^c^
Do you know at least 2 risk factors of BC?		
Yes^a^	25(5.8)	23(9.8)
No	408(94.2)	211(90.2)
Do you know at least 2 signs or symptoms of BC?		
Yes^b^	115(26.6)	93(39.7)
No	317(73.4)	141(60.3)
Which of the following methods are used to diagnosis BC?		
Physical exam	44(10.4)	25(11.2)
Imaging/x-ray	39(9.2)	23(10.3)
Biopsy	1(0.1)	1(0.1)
I don’t know	341(80.2)	175(78.1)
Which of the following methods are used to screen for BC?		
Mammography	40(9.2)	32(13.8)
Regular CBE	47(10.8)	41(17.7)
Monthly BSE	39(8.9)	31(13.4)
I don’t know	310(71.1)	128(55.2)
Do you know how to do BSE?		
Yes	237(54.4)	
No	199(45.6)	NA
Have you ever had a CBE done by a clinician?		
Yes	28(6.4)	NA
No	408(93.6)	
Have you ever had a CBE done by a traditional healer?		
Yes	2(6.9)	NA
No	27(93.1)	

*NA* Not applicable to male respondents, *BC* breast cancer, *BSE* breast self-exam, *CBE* clinical breast exam

^a^respondent can answer at least 2 of 15 yes/no questions about potential risk factors correctly (9 yes, 6 no)

^b^respondent can answer at least 2 of 4 yes/no questions about potential signs and symptoms correctly (3 yes, 1 no)

^c^ total numbers may not add up to 442 (females) or 237 (males) because some respondents did not respond to specific questions

When asked what they would do if they noticed a breast lump or swelling, over 90% of the women reported they would want to visit a health facility within a week (Table 4). Forty-nine percent of women indicated that their husband would determine where they would seek care.

**Table 4:** Breast health seeking behaviour and practice among female members of households in Kaloleni sub-county, Kenya

Question	n(%)^a^
Where would you go if you have a breast swelling?	
Health facility	418(95.4)
Faith healer	3(0.7)
Traditional healer	2(0.5)
Other	1(0.2)
Don’t know	14(3.2)
How soon would you seek help for a breast lump?	
< 1 week	370(90.7)
< 1 month	27(6.6)
1–3 months	8(2.0)
Depends on factors	3(0.7)
Who decides where you would seek help for a breast problem?	
Husband	213(49.0)
Myself	129(29.7)
Parents	54 (12.4)
In-laws	3(0.7)
Other	36(8.3)
What health facility would you go to to seek help for a breast problem?	
Dispensary	217(51.2)
Health centre	69(16.3)
Sub-county hospital	105(24.8)
County or Teaching & Referral hospital	33(7.8)
Why would you choose this facility?	
Location close to home	238(55.4)
Quality of service	131(30.5)
Finances	11(2.6)
Other (family or husband’s decision, transportation, etc.)	60(11.7)
Would you allow a male doctor to examine your breast?	
Yes	354(82.3)
No	69(16.1)
Would you allow a male traditional healer to examine your breast?	
Yes	76(17.4)
No	357(81.5)

^a^ total numbers may not add up to 442 because some respondents did not respond to specific questions

Most women reported they would go to a lower level health facility, either a dispensary (51%) or health centre (16%). For 55% of the women, the choice of facility would be influenced by proximity to their home (Table 4).

Over 90% of the heads of households said that their wives shared their health concerns with them, and that they enquired about why their wives visited health facilities (Table 5). Ninety-four percent of male heads of households indicated that they would decide whether and where their wives would seek help for a breast lump. More than 90% of the men said they would encourage and support their wives to seek help at a health facility within a week if they had breast lump. Over 85% of men would be comfortable having their wives examined by a male health care worker, but only 20% would allow their wives to be examined by a male traditional healer. Over 90 % of men stated they would support their wives if they were diagnosed with breast cancer (Table 5).

**Table 5:** Male heads of households’ practice and support of female members’ breast health-seeking behavior in Kaloleni sub-county, Kenya

Question	n(%)ǂ
Does your wife tell you of her health concerns (*n* = 221)	
Yes	212(95.9)
No	9(4.1)
Do you enquire why wife visits health facility (*n* = 216)	
Yes	200(92.6)
No	16(7.4)
If you knew that BC could be detected at an early stage, would you encourage your wife to get screened?	
Yes	220(97.4)
No	
Who would pay for the screening tests?	
Myself	201(92.6)
Her family	6(2.8)
My parents	4(1.8)
Other	6(2.8)
What would be your role if your wife had a breast problem?	
Ask her to go to the hospital	219(94.0)
Other	3(1.3)
Not my concern	11(4.7)
Who would decide where your wife would seek help?	
Myself	222(96.5)
My parents	5(2.2)
Her parents/ Community elders/others	3(1.2)
Where would you take her if your wife had a breast lump?	
Health facility	224(96.1)
Faith healer/ Traditional doctor	7 (3.1)
What health facility would you take her to to seek help for a breast problem?	
Dispensary	91(40.3)
Health centre	40(17.7)
Sub-county hospital	49(21.7)
County or Teaching & Referral hospital	46(20.4)
Why would you choose a particular facility?	
Location close to home	221(94.4)
Husband’s/family decision	13(5.5)
How soon would you seek help if your wife had a breast lump?	
< 1 week	119(93.0)
< 1 month	4(3.1)
Other	5(4.0)
Would you allow a male doctor to exam your wife’s breast?	
Yes	201(86.3)
No	31(13.3)
Would you allow a male traditional healer to exam your wife’s breast?	
Yes	47(20.1)
No	185(79.1)
Who would you tell if your wife had a BC diagnosis?	
Immediate family	179(77.8)
Community, elders, religious leaders, others	27(11.7)
No one	24(10.4)
What would you do if wife was living with BC	
Support her	211(91.0)
Take another wife	13(5.6)
Leave her, don’t know, other	6(2.6)
Who would support you wife if she had BC?	
Myself	176(75.5)
Our children	13(5.6)
Wife’s family	7(3.0)
Community	1(0.4)
Other	36(15.5)

*BC* breast cancer

ǂ totals do not add upto 237 because some respondents did not respond to specific questions

### Qualitative findings

Each Focus Group Discussion was made up of 6–7 participants; a total of 2 Medical Officers, 2 Clinical Officers, 16 Nurses and 2 technologists participated in the one-on-one Key Informant Interviews. Six themes emerged from the analysis of these discussions as barriers to accessing breast cancer care in Kaloleni.

#### Poor knowledge of the signs of breast cancer

Among the focus group participants, knowledge about the signs of breast cancer varied. Many described having wounds that do not heal and that have a foul smell or holes, and some talked about the breast “rotting”, while others mentioned various words that referred to lumps (hard balls, cones, swelling, *tezi* in the Giriama language). Itching (*kukaka* in Giriama), pain and discharge from wounds were also mentioned as signs a woman may have breast cancer. The participants’ opinions about the causes of breast cancer were also wide-ranging, with some being accurate and others not being correct. One community member linked cancer to poor hygiene, another to being pierced by something sharp during a forest walk, and yet another attributed it to malnutrition. A community member recommended a community-level solution “*to educate the community in general that if one makes a good follow-up they can get healed.*”

#### Fear of stigma associated with breast cancer diagnosis, and limited decision-making autonomy among women

Women might fear a diagnosis of breast cancer due to the potential of being considered undesirable, being cast off by husbands, and potentially being replaced. A village chief noted, “*Maybe if a girl is known to have breast cancer, one may be divorced if she is married. If a girl gets cancer and one of her breasts is removed obviously that one will be rejected.* [Men] *may get married to other women. One time I handled such a case. A woman had cancer. The husband’s family did not want her… They disowned her.”*

Social rejection or stigma was a barrier mentioned by a few individuals in relation to the reluctance to receive a breast cancer diagnosis or accept indicated interventions such as mastectomies. One female community member described her neighbour: “*She has two growths on the breast and one under the tongue. The thing that makes her not go* [for medical attention] *is* [the fear of] *being despised.”* It is even more difficult for unmarried women: “*Many women here at our place do not have a husband…if you don’t have someone to support you then you will die.”*

Male heads of households pointed out that they generally felt left out of breast health conversations. One participant described “*our sisters don’t tell us anything about breasts. Whatever they are told, when they get back home they hide it*.”

There was encouragement among heads of households to become more involved in their wives’ breast health. One head of household recommended, “*If your wife complains of any problem, don’t deny her the opportunity to go to hospital; listen to her and assist her. Us men should give the necessary support to our wives.”* Another participant recommended, “*The husband must go to the doctor to be given information about his wife’s condition and use of drugs.*”

From the health care worker’s perspective, limited decision-making autonomy among women was thought to influence their ability to seek care at health facilities. Many said that women must get the permission of their husband, or their mother-in-law in their husband’s absence, to visit health facilities. One health care worker explained that reliance on husbands was particularly strong among “*those less educated*”.

Health care workers also perceived discomfort with clinical breast examinations among both men and women, particularly those performed by male health care workers. Some health care workers described husbands as “*obstacles, thinking* [their wives] *are going to expose their private parts to other people.*”

A health care worker recommended “awareness creation” among both women and men as a solution to engaging heads of households and women in health care decision-making and feeling more comfortable with the clinical breast exam, noting “*Sensitisation should be done to enable* [heads of households] *to allow their wives to go for check-ups.”*

#### Preference for traditional healers

A preference by some families for traditional healers was described, especially among families which thought the cause of breast cancer involved witchcraft or curses*.* One female participant referred to a community member who had breast cancer but the family felt that she was *“bewitched”. “So instead of taking her to the hospital, they took her to a traditional healer.”* According to one health worker, these explanatory models are “*especially a belief in some diseases which the community are not so much aware about*”. Health-seeking behaviours centred on self-medicating or going to traditional healers first were most common when symptoms were not considered serious enough to warrant a visit to the health facility. In many instances, there was a perception among health care workers that a lack of awareness of the signs and symptoms of breast cancer led women to seek care from an “*alienda kupigwa maji ya kiapo* [a phrase in Kiswahili meaning going to a traditional healer] *instead of going to a hospital*”.

Both community members and healthcare providers described the use of traditional healer services by some residents as a deterrent to timely access of medical services, resulting in women presenting at the health facilities at later, less curable stages of breast cancer. In some cases, the choice to use traditional medicine was simply driven by affordability and their proximity to the patient’s home. One health care worker mentioned “*if there is a traditional healer nearby, they find it easier to go to him than coming to a hospital. Long distance makes them not come to health facilities.*”

#### Lack of trust in the health care system

There was a difference in the perception of the level of trust in the health care system among community members and health care workers. Some community members had more faith in traditional healers, noting “*Some still believe in herbal medicine and some have woken up a little and go to hospital. They will only come when the traditional healers fail to treat them*.*”*

Instances of suspicion about the real objective of a community outreach activity related to breast cancer were also described, “*They say medicines given from hospital here* [to the community] *could be family planning pills given secretly, and many of them are not ready to do family planning. Others even say that* [community outreach] *is an organization for devil worshippers*.”

In contrast, health workers mostly felt that the community generally trusted the health care system. The evidence for this in their minds came from the large numbers seeking assistance at the health facility without much prompting, especially for antenatal care or family planning services, and because of the availability of qualified staff.

#### Inadequate access to services

A lack of adequate breast cancer services accessible to communities, both in terms of cost and distance, was repeatedly acknowledged by health workers and complained about by the community. These inadequate services ranged from early detection interventions to treatment. One female community member noted, “*They don’t go for screening because we don’t have a doctor who can offer that service.”* Availability of mammography services was also limited and many health workers were not sure where a woman would need to go to receive mammography. A health care worker noted “*our health facilities are not equipped with the necessary equipment, so even if they come they may be referred which is very complicated for them.”*

Long distances from homes to facilities which have recommended breast cancer treatment services also pose a big challenge to the community. Some treatment and surgical interventions were only available in Mombasa town, which is fifty kilometres by road from Kaloleni, or in the capital Nairobi, which is much further away. This is compounded by an inability to afford the necessary means of transportation, and it is exacerbated by poor road conditions made impassable at times during heavy rains. When asked about access barriers, both community members and health workers cited distance as a significant barrier for the community. A community member mentioned, “*Someone will feel it’s better to die from home than going there. The services are very far; they need to come closer to the people.”*

Poverty was also cited as a major barrier to accessing health care services among Kaloleni residents. Financial constraints and the ability to afford specialised treatment offered at referral centres was cited as a particular challenge. One health care worker noted “*There is also the fear of being referred to Kenyatta National Hospital, as people do not have the money required for treatment there*.”

Poor access to treatment seems to have led to a perception that cancer is essentially fatal, which also contributes to treatment non-compliance for those who were diagnosed with breast cancer.

#### Limited early-detection services and patient management guidelines

Early detection programmes were not offered at many of the health facilities, and they were not integrated into other programmes where attendance was high, such as antenatal or family planning clinics, at any of the facilities surveyed.

Most of the health care workers interviewed said their facilities had no tracking system for women who required mammography or had received a breast cancer diagnosis that required treatment. Most of the facilities engaged the volunteer community health volunteers (CHWs) to follow up women who did not go for treatment. Health care workers suggested having a specific office for breast cancer to coordinate activities at a national level. One expressed frustration with the lack of staffing and management guidelines: “*First, as I said, we need a* [national breast cancer] *centre.* [Second] *we need more trained personnel. Thirdly, if we have a TB program, an HIV program, and a malaria program, we also need a cancer program*.*.. Even guidelines, we have guidelines for malaria, TB and HIV, so why can’t we have guidelines for cancer? If we now get a suspected cancer patient, we don’t have a protocol to guide us*.”

One health care worker underscored the importance of decentralizing services, coordination and programme planning to ensure that there is advocacy for cancer services down to the sub-county level, “*I would push to see that the Reproductive Health Bill also includes promotion of health-seeking behaviour for breast and cervical cancer. And also, the Department of Non-communicable Diseases should be activated down to the sub-county level*.”

## Discussion

Our mixed methods study assessed the breast cancer knowledge, perceptions and practices of women, male heads of households, community opinion leaders, and health care workers in a rural coastal community in Kenya and describes gender-based and structural barriers that prevent women from accessing breast cancer care. To our knowledge, this is the first study from Kenya that also provides insight into the male heads of households’ knowledge of breast cancer and their role in influencing their wives’ access to breast health care.

Nearly half of the women in this study had no formal education, and nearly all of the others had only attended primary school. Most had heard of breast cancer, and knew it was a serious disease, but only a quarter knew any of its signs or symptoms, and only 6% had ever had a clinical breast examination performed by a clinician. Over 90% of the women said they would go to a health facility within a week if they felt a breast lump, but most said that the decisions about whether and where to go would be made by their husbands or other family members.

The male heads of households were better educated, with 80% having had formal education. Over 90% said they knew that breast cancer was a serious disease, and they would take their wife to a health facility within a week if she had a breast lump. They also said they would support her, and not leave her, if she had breast cancer.

The Focus Group Discussions and Key Informant Interviews confirmed that the population, its opinion leaders, and even some of its health care workers had a poor knowledge of the signs and symptoms of breast cancer. In contrast to the quantitative survey results, however, the opinion leaders and health care workers believed strongly that stigma, social rejection, and fear of losing their husbands were important reasons for women to hide or delay seeking help for breast lumps, leading to later breast cancer diagnoses and poorer outcomes. They also felt that using traditional healers was much more common than the quantitative surveys would suggest, reflecting both the population’s ignorance of breast cancer signs and the individual’s hope that the symptoms were due to a less important, less stigmatizing problem, again leading to delayed diagnosis and treatment. The FGDs and KIIs also identified lack of trust in the health care system, inadequate access to services, and lack of standard breast cancer management guidelines throughout the healthcare system as reasons for late diagnosis, suboptimal care and poor outcomes.

There was evidence from the qualitative study that late diagnoses are common. Many of the survey respondents and members of the various focus groups defined breast cancer as a “wound” of the breast. This provides indirect evidence that, similar to most LMICs [10, 11], including Kenya [2], breast cancer patients in Kaloleni probably present at an advanced stage of disease when the tumour has ulcerated through the overlying skin. Moderate level of evidence suggests a link between delay in patient presentation with various factors that include, lack of breast cancer awareness, alternative treatment use, and rural residency among others [12].

Cultural, traditional and religious beliefs are some of the factors contributing to women not seeking breast cancer services from the health facilities. Key barriers to accessing breast health care were the lack of awareness of the risk factors, signs and symptoms of BC; the screening, diagnosis and treatment options for the disease; and access to accurate information. It is interesting to note that health workers also admitted they had inadequate knowledge about BC. They expressed a desire to be provided more educational and training opportunities about BC screening, diagnosis and treatment. This is not unique to Kaloleni; published data from a Nigerian teaching hospital reported that irrespective of age and professional qualification, more than 40% of the115 nurses who participated in their study regarded pain as a sign of early BC [13]. Similarly, a recent review article from India [14], reported that irrespective of their socio-economic and educational background, among the 7066 women aged 15–70 years surveyed, there was no improvement in the knowledge of risk factors for BC over an 8 year period; respondents demonstrated varying levels of awareness of risk factors such as family history (13–58%), reproductive history (1–88%) and obesity (11–51%) [14].

Efforts to improve breast health care requires the recognition of the important role health workers play in public education [15]. Community opinion leaders and health care providers can play a key role in educating and referring women for screening. Any proposed BC educational and training activity should therefore engage and target community leaders and various cadres of health professionals to ensure cascading of accurate information about BC at the community level and the public at large.

In our study, the data sources and the data collection methods for the quantitative and qualitative components differed, which may help explain some of the differences in the findings of these two components. The quantitative component consisted of interview-administered questionnaire surveys of female members and male heads of households, while the qualitative component consisted of group discussions with community leaders and relatively unstructured discussions with individual health care workers. Social desirability bias could have been a factor in the questionnaire surveys, especially because the questionnaires were administered by community health workers who were members of the community and were well known to those being interviewed. There were also differences seen in the results along gender lines. As noted above, the perception among health care workers, some heads of households, and FGDs of women was that there is often a lack of support for women with breast cancer from male heads of households. However, our survey findings and the FGD groups of men indicated that men would be financially and socially supportive of women in their household who were diagnosed with breast cancer. These discordant findings may be partly due to men answering questions the way they thought they “should” answer them, but it also may indicate that the real barrier may not be the men’s attitudes but the women’s perception of the men’s attitudes, and their (unfounded?) fear of rejection if their male partner found out about their cancer. This has important implications in terms of developing content for awareness programs. It is also important that some village chiefs and heads of households described a desire to be more involved in the breast health care of their wives and female cohabitants.

Perhaps the quantitative data and the openness expressed by some heads of households in the qualitative study reflect an opportunity for engagement of heads of households in breast health, either through community outreach or the CHWs. The role of husbands as a barrier to early detection practices has previously been reported; young Kamba women with breast cancer risked being abused and ostracized by their husbands [16], and the subservient role of women in many societies leads to denial of symptoms and delay in diagnosis [17]. There needs to be a continued commitment by civil society and governments to address issues related to the lack of female autonomy, lack of social support or social capital, and marginalization [18]. In addition, enhanced engagement of men in health decision making and use of study models that are sensitive to gender and household dynamics may support the male role in health decisions that pertain to women’s health, including breast cancer [19].

According to the quantitative results there was little interest among community members in using traditional healers to manage breast lumps; however the qualitative interviews with women, men, and health care workers described a much more significant role for these healers in responding to breast symptoms and in provision of health care in general. The role of traditional healers in breast cancer detection and treatment should be explored further, to understand whether they are, in fact, associated with delayed diagnosis and treatment in rural African communities. This has been previously researched in other geographical areas, and this belief has been identified among Chinese and South Indian women [20]. A complex interplay of cultural, spiritual, and individual experiences and perceptions results in traditional and spiritual healers often being the first port-of-call for women with breast symptoms [21]. Extending educational activities to and encouraging participation of traditional and spiritual healers in the implementation of local breast cancer control programs can be a bridge to build trust and mutual understanding among the various health sectors, potentially encouraging traditional healers to refer patients with breast cancer symptoms more rapidly to government health care facilities.

Structural barriers such as long distances to health facilities, lack of affordable services, lack of equipment to offer screening or treatment, lack of education about cancer, and minimal sensitization of both women and men to the symptoms of breast cancer and the options for diagnosis and treatment were cited as barriers to uptake of these services. Inadequate diagnostic facilities, requiring patients to travel long distances, and the costs of transportation, as well as lack of governmental support for screening, diagnosis, and treatment costs, which were all mentioned by our study participants, have previously been reported as major deterrents in getting timely advice and treatment for breast cancer [22].

Poverty constitutes the underlying common denominator and most important barrier contributing to lack of awareness and delayed patient presentation. Evidence from multiple studies show that poverty is manifested by lower income, lower education level, rural residency, and lack of access to healthcare systems [12]. In Kilifi County within which Kaloleni is located, the poverty rate is 70.8%, significantly higher than the national average of 45.9% [8]. In 2005, the World Health Organization (WHO) member states endorsed a resolution to advocate for the adoption of universal health coverage and proposed health financing systems that can alleviate barriers to access to health care [23]. However, there are multiple factors to consider, and financial solutions alone will not ensure access to health services [18].

The use of the Community Health Workers (CHWs) was repeatedly mentioned in our focus group discussions as a good avenue to extend educational activities which are more acceptable to the community. Community education and continuous sensitization to the signs and symptoms of breast cancer, the fact that early detection increases the chances for cure, and the options for screening, diagnosis and treatment will be key to empowering people with accurate knowledge and dispelling some of the myths about breast cancer, which will encourage women to seek breast health services in a timely manner. Organized outreach breast cancer screening activities involving the CHWs would also enable these volunteers to reach remote populations within their units better. Lessons could be learnt from The Deep South Network for Cancer Control (DSN), a community–academic partnership operating in Alabama and Mississippi [24], which formulated a community action plan to address cancer disparities through focusing on participation of local communities in education, research, and training. Some of the objectives were to increase cancer screening through raising awareness at the individual, provider, and system levels and training community partners to become effective advocates. The guiding principle and success of this plan depended upon trust, respect, and an appreciation of partners’ strengths and differences [25].

One strength of this study was the combined quantitative and qualitative approach, which allowed additional insights to be revealed when the data from the two approaches were compared. Other strengths were the questioning of the male heads of households and the coordinated questioning of women, men, community leaders and health care professionals.

There were also several limitations to our study. As with most survey-based studies, our quantitative data could have been limited by selection bias and social desirability bias, especially since the surveys were administered by CHWs (community members) who served as the interviewers. The interpretation of the findings of our qualitative research may also have been limited by research bias, although we tried to mitigate this by involving a multidisciplinary team of researchers in this analysis. Finally, this study was confined to a small geographic region of rural Kenya, which may limit the generalizablility of the findings to similar low resource settings.

## Conclusion

Determining the level of breast cancer knowledge and the perceived barriers to accessing breast cancer care are the first steps in establishing locally relevant intervention programs to reduce the burden of this disease. A clear message from this study is the need for improved and coordinated breast cancer education in all groups in the society, including women, men, community leaders and all levels of health care workers. Future research should be encouraged to identify successful strategies to achieve this goal in poorly educated patriarchal rural communities like Kaloleni. There is also a great need for standardized patient management guidelines, more widespread and decentralized early detection and diagnostic services, and better access to necessarily centralized treatment services.

## Additional files

Additional file 1: Male questionnaire (DOCX 35 kb)

Additional file 2: Female questionnaire (DOCX 35 kb)

Additional file 3: Focus group discussion guide. (DOCX 16 kb)

Additional file 4: Key Informant interview guide (DOCX 30 kb)

Additional file 5: Female questionnaire dataset (XLSX 112 kb)

Additional file 6: Male questionnaire dataset (XLSX 72 kb)

## Abbreviations

AKU - EA: Aga Khan University - East Africa
BC: Breast Cancer
CEW: Community Extension Worker
CHW: Community Health Worker
CU: Community Unit
FGD: Focus Group Discussions
IPHC: Integrated Primary Health Care
KII: Key Informant Interviews
LMIC: Low and Middle-Income Countries
RAP: Rapid Assessment Process
REACH: Racial and Ethnic Approaches to Community Health
UCSF: University of California San Francisco
