# Supplementary material for: Breast Cancer knowledge, perceptions and practices in a rural Community in Coastal Kenya
Source: BMC Public Health. 2019 Feb 12;19:180. doi: 10.1186/s12889-019-6464-3 (PMC6373063; doi:10.1186/s12889-019-6464-3)
Supplement: Supplementary file 1 — Male questionnaire (DOCX 35 kb) [file 12889_2019_6464_MOESM1_ESM.docx]

| \|  \|  \| \| --- \| --- \| \| Name and Signature of Person conducting informed consent \| ***Date*** \| \|  \|  \| | | | | | | | | | | | | | | | | | | | | | | | | | | |
| --- | --- | --- | --- | --- | --- | --- | --- | --- | --- | --- | --- | --- | --- | --- | --- | --- | --- | --- | --- | --- | --- | --- | --- | --- | --- | --- | --- | --- | --- | --- | --- | --- |
| **Aga Khan University Hospital, Nairobi**  **Breast Cancer KAP Questionnaire** | | | | | | | | | | | | | | | | | | | | | | | | | | |
| Start time | | |  |  |  |  |  | **Questionnaire code-:** | | | | | | | | | | | | |  |  |  | |  | |
|  | | | | | | | | | | | | | | | | | | | | | | | | | | |
| **CLUSTER NO.** | | |  |  |  |  |  | Household No | | | | | | | | | | | | |  |  |  | |  | |
| Village: | | | | | | | | | | | | | | | | | | | | | | | | | | |
| Name of household head | | | | | | | | | | | | | | | | | | | | | | | | | | |
| Relationship to Respondent  1=Husband***;*** 2=Brother***;*** 3=Father***;*** 4=Uncle***;*** 5=Grandfather***;*** 6=Other (specify) ***________________*** | | | | | | | | | | | | | | | | | | | | | | | |  | | |
| **Introduction** | | | | | | | | | | | | | | | | | | | | | | | | | | |
| Researchers at The Aga Khan University Hospital, Nairobi are interested in learning how we can improve women’s health in Kaloleni sub-county, Kilifi County particularly as it relates to breast cancer. Breast Cancer involves a growth in the breast that has the ability to spread outside the breast to other parts of the body, and is a public health problem that afflicts women all around the world. In Kenya, breast cancer is the second most common cancer in women after cancer of the cervix. We are interviewing 450 women, ages 15 and above and 250 men from randomly selected households within Kaloleni, to gain a greater insight as to women’s current breast cancer knowledge, attitudes, and beliefs. The collective outcomes of our interview will be used to inform policy on promotion of early detection and timely treatment of breast cancer and establish an appropriate population based breast cancer screening and awareness program Your contribution will be important to conduct this study and your participation will be kept confidential. Before we begin, can you confirm your eligibility to take part in the interview by answering three questions:  Name :_________________________________**DoB: DD/MM/YY___________________________**  Mobile number: **________________________** | | | | | | | | | | | | | | | | | | | | | | | | | | |
| 1.0 Questions on Socio demographic Factors | | | | | | | | | | | | | | | | | | | | | | | | | | |
| **No.** | **Question** | | | | | | | | | **Code** | | | | | | | **No.** | **Question** | | | | | | | | **Code** |
| 1.1 | Total Number of Household Members living together | | | | | | | | |  | | | | | | | 1.2 | Marital status   1. Single 2. Married 3. Divorced 4. Widowed 5. Other : specify | | | | | | | |  |
|  |  |  |  |  |  |  |  |  |  |  | | | | | | |  |  |  |  |  |  |  |  |  |  |
| 1.3 | Highest level of Education   1. None 2. Primary 3. Secondary 4. Tertiary 5. Education classes for Mature students 6. Declined to answer | | | | | | | | |  | | | | | | | 1.4 | Religion   1. Catholic 2. Protestant 3. Islam 4. Traditional 5. Other   ------------------------------------   1. None 2. Declined to answer | | | | | | | |  |
| 1.5 | Occupation   1. Clerical/Management/Admin 2. Crafts 3. Trader 4. Professional technical 5. Farmer 6. Housewife 7. Other – specify 8. None | | | | | | | | | | | | | | | | | | | | | | | | |  |
| 2.0 Questions Addressing General Knowledge on Breast Cancer | | | | | | | | | | | | | | | | | | | | | | | | | | |
| 2.1 | Have you heard the word/term Cancer before this survey?   1. Yes 2. No | | | | | | | | | |  | | 2.2 | | | | | Have you heard the word/term breast cancer before this survey   1. Yes 2. No | | | | | | | |  |
|  |  |  |  |  |  |  |  |  |  |  |  | |  |  |  |  |  |  |  |  |  |  |  |  |  |  |
| 2.3 | Have you ever known a person suffering from breast cancer   1. Yes 2. No ***-If no skip to 2.5*** | | | | | | | | | |  | | 2.4 | | | | | If Yes who?   1. Friend 2. Mother 3. Sister 4. Daughter 5. Other   ---------------------------------------------- | | | | | | | |  |
|  |  |  |  |  |  |  |  |  |  |  |  | |  |  |  |  |  |  |  |  |  |  |  |  |  |  |
|  |  | | | | | | | | | |  | |  | | | | |  | | | | | | | |  |
| 2.5 | How much do you know about breast cancer     1. Nothing at all 2. I know a little about the disease 3. I am very familiar with it 4. I have only heard the term before 5. Declined to answer | | | | | | | | | |  | | 2.6 | | | | | What do you know about Breast cancer? Is it a serious illness?   1. Yes 2. No 3. Don’t know     *(****only answer this if 2.5 is answered as option 2- 4)*** | | | | | | | |  |
|  |  |  |  |  |  |  |  |  |  |  |  | |  |  |  |  |  |  |  |  |  |  |  |  |  |  |
| 2.7 | Do you know what the cause of breast cancer is? [more than one answer can be selected]   1. A Virus 2. Close contact with person with breast cancer 3. Hereditary (passed from parent to child) 4. Lifestyle choices (foods, smoking, lack of exercise, etc.) 5. Evil eye 6. Witchcraft 7. No-one knows the cause/ 8. I don’t know 9. Declined to answer 10. Other - specify   -------------------------------------------- | | | | | | | | | |  | | 2.8 | | | | | Do men get breast cancer   1. Yes 2. No 3. Don’t know | | | | | | | |  |
|  |  |  |  |  |  |  |  |  |  |  |  |  |  |  |  |  |  |  |  |  |  |  |  |  |  |  |
| 2.9 | Do you think breast cancer is a curable disease?   1. Yes 2. No 3. Don’t know | | | | | | | | | |  | | 2.10 | | | | | Do you think it is possible to survive breast cancer if detected early?     1. Yes 2. No 3. Don’t know | | | | | | | |  |
|  |  |  |  |  |  |  |  |  |  |  |  | |  |  |  |  |  |  |  |  |  |  |  |  |  |  |
|  |  | | | | | | | | | |  | |  | | | | |  | | | | | | | |  |
| 2.11 | Do you think breast disease can be treated by traditional healer?   1. Yes 2. No 3. Don’t know | | | | | | | | | |  | | 2.12 | | | | | Do you know the signs and symptoms of breast cancer?     1. Yes 2. No **[Skip to 2.14]** | | | | | | | |  |
|  |  |  |  |  |  |  |  |  |  |  |  | |  |  |  |  |  |  |  |  |  |  |  |  |  |  |
| 2.13 | If Yes in **Q2.12**, please list at least three signs and symptoms  **--------------------------------------------------------------------------------------------------------------------------**  **-------------------------------------------------------------** | | | | | | | | | |  | | 2.14 | | | | | Do you know how breast cancer is diagnosed?     1. Yes 2. No **[Skip to 3.1]** | | | | | | | |  |
|  |  |  |  |  |  |  |  |  |  |  |  |  |  |  |  |  |  |  |  |  |  |  |  |  |  |  |
|  |  |  |  |  |  |  |  |  |  |  |  |  | 2.15 | | | | | **If Yes [select one or more response]**     1. Imaging (X ray of breast) 2. Physical exam by doctor –examination of a doctor 3. Biopsy –(Tissue from breast) 4. Other: specify 5. Don’t know 6. Declined to answer | | | | | | | |  |
|  |  |  |  |  |  |  |  |  |  |  |  |  |  |  |  |  |  |  |  |  |  |  |  |  |  |  |
| 3.0 Questions on Knowledge of Risk Factors, Symptoms, Screening Methods | | | | | | | | | | | | | | | | | | | | | | | | | | |
| 3.1 | Are the following sign and symptoms of breast cancer? | | | | | | | | | | |  | | | 3.1a | | | Lump in breast   1. Yes 2. No 3. I don’t know 4. Declined to answer | | | | | | | |  |
|  |  |  |  |  |  |  |  |  |  |  |  |  | | |  |  |  |  |  |  |  |  |  |  |  |  |
| 3.1b | Pain in breast   1. Yes 2. No 3. I don’t know 4. Declined to answer | | | | | | | | | | |  | | | 3.1c | | | Nipple discharge   1. Yes 2. No 3. I don’t know 4. Declined to answer | | | | | | | |  |
|  |  |  |  |  |  |  |  |  |  |  |  |  | | |  |  |  |  |  |  |  |  |  |  |  |  |
| 3.1d | Painless breast lump   1. Yes 2. No 3. I don’t know 4. Declined to answer | | | | | | | | | | | | | | | | | | | | | | | | |  |
|  |  |  |  |  |  |  |  |  |  |  |  |  |  |  |  |  |  |  |  |  |  |  |  |  |  |  |
| 3.2 | Do you know the risk factors of breast cancer [Ask against each item] Code response as either**:**     1. Yes 2. No 3. I don’t know 4. Declined to answer   ***If 3.2 is 2,3 or 4 skip to 3.3*** | | | | | | | | | | | | | | | | | | | | | | | | | |
| 3.2a | Advanced age   1. Yes 2. No 3. I don’t know 4. Declined to answer | | | | | | | | | |  | | | | 3.2i | | | | | Early menarche   1. Yes 2. No 3. I don’t know 4. Declined to answer | | | | | |  |
| 3.2b | Poverty   1. Yes 2. No 3. I don’t know 4. Declined to answer | | | | | | | | | |  | | | | 3.2i | | | | | Late menopause   1. Yes 2. No 3. I don’t know 4. Declined to answer | | | | | |  |
| 3.2c | Wearing brassieres   1. Yes 2. No 3. I don’t know 4. Declined to answer | | | | | | | | | |  | | | | 3.2k | | | | | Use of oral   1. Yes 2. No 3. I don’t know 4. Declined to answer | | | | | |  |
| 3.2d | First child bearing after 30 years   1. Yes 2. No 3. I don’t know 4. Declined to answer | | | | | | | | | |  | | | | 3.2l | | | | | Use of tobacco/ tobacco products   1. Yes 2. No 3. I don’t know 4. Declined to answer | | | | | |  |
| 3.2e | Obesity   1. Yes 2. No 3. I don’t know 4. Declined to answer | | | | | | | | | |  | | | | 3.2n | | | | | Use of alcohol/ traditional brew   1. Yes 2. No 3. I don’t know 4. Declined to answer | | | | | |  |
| 3.2f | Contact with breast cancer patient   1. Yes 2. No 3. I don’t know 4. Declined to answer | | | | | | | | | |  | | | | 3.2o | | | | | A high fat diet   1. Yes 2. No 3. I don’t know 4. Declined to answer | | | | | |  |
| 3.2g | First relative positive family history   1. Yes 2. No 3. I don’t know 4. Declined to answer | | | | | | | | | |  | | | | 3.2p | | | | | Big breasts   1. Yes 2. No 3. I don’t know 4. Declined to answer | | | | | |  |
| 3.2h | Long lactating period   1. Yes 2. No 3. I don’t know 4. Declined to answer | | | | | | | | | | | | | | | | | | | | | | | | |  |
| 3.3 | Do you know the Screening Methods for early detection of breast cancer?   1. Yes 2. No **[Skip to 4.1]** | | | | | | | | | | | | | | | | | | | | | | | | |  |
| 3.3a | Others - :specify________________________________________________________________________ | | | | | | | | | | | | | | | | | | | | | | | | |  |
| 3.4 | Monthly breast self-examination     1. Yes 2. No | | | | | | | | | | | | | | | | | | | | | | | | |  |
| 3.5 | Regular clinical breast exam (CBE) once a year   1. Yes 2. No | | | | | | | | | | | | | | | | | | | | | | | | |  |
| 3.6 | Mammography/imaging in health center or hospital   1. Yes 2. No | | | | | | | | | | | | | | | | | | | | | | | | |  |
| 4.0 Questions on Practice and Barriers to Practice | | | | | | | | | | | | | | | | | | | | | | | | | | |
| Breast self-examination is a method used where women can check their own without needing to see a doctor | | | | | | | | | | | | | | | | | | | | | | | | | | |
| 4.1 | | Would you allow your wife to be examined by:/***Male-Traditional Healer***   1. Yes 2. No 3. I don’t know 4. Declined to answer | | | | | | | | |  | | | 4.2 | | | | Would you allow your wife to be examined by: ***Health-Care worker***   1. Yes 2. No 3. I don’t know 4. Declined to answer | | | | | | | |  |
|  |  |  |  |  |  |  |  |  |  |  |  | | |  |  |  |  |  |  |  |  |  |  |  |  |  |
| 4.3 | | Would you allow your wife to be examined by: ***Male Doctor***   1. Yes 2. No 3. I don’t know 4. Declined to answer | | | | | | | | | | | | | | | | | | | | | | | |  |
|  |  |  |  |  |  |  |  |  |  |  |  |  |  |  |  |  |  |  |  |  |  |  |  |  |  |  |
| 5.0 Questions Addressing Health Seeking Behaviour and Perceived Barriers | | | | | | | | | | | | | | | | | | | | | | | | | | |
| 5.1 | | If your wife has a health concern does she always tell you the details?   1. Yes 2. No | | | | | | |  | | | | | | | 5.2 | | | Do you normally enquire why your wife visited the hospital?   1. Yes 2. No | | | | | | |  |
| 5.3 | | What would be your role if your wife had a breast problem?”   1. Not my concern 2. Will ask her to go to hospital 3. Will ask her to consult other women 4. Other – Specify   --------------------------------------------- | | | | | | |  | | | | | | | 5.4 | | | What would you do if your wife or close female relative in your family is discovered with breast swelling lump?   1. Health Facility 2. Faith Healer 3. Traditional Doctor 4. Stay at home 5. Self-medication 6. Don’t know 7. Other | | | | | | |  |
| 5.4a | | Would you consider taking another woman/ ***wife if your wife has breast cancer***   1. Yes 2. No | | | | | | | | | | | | | | | | | | | | | | | |  |
| 5.5 | | If you do not choose to go to a health facility what would be the reason/s   1. Shortage of doctors 2. No medicines 3. Rude/uncaring ***staff*** 4. Long queues 5. Too expensive 6. It’s God’s will 7. Other – specify   -------------------------------------------------- | | | | | | |  | | | | | | | 5.6 | | | Who makes the decision as to where she will seek assistance   1. Myself 2. The patient herself 3. My parents 4. Religious leaders 5. Other –specify   ---------------------------------------- | | | | | | |  |
| 5.7 | | Which health facility would you go to seek assistance in the event of having a breast problem? **(Only answer if question 5.1 is 1)**   1. Dispensary 2. Health centre 3. Sub-county hospital 4. County hospital 5. Teaching and referal hospital | | | | | | |  | | | | | | | 5.7a | | | What would be the reason for the choice above?   1. Vicinity to facility 2. Husband 3. Family decision 4. Finances 5. Transportation 6. Quality of service   Other*)* | | | | | | |  |
| 5.8 | | If your wife or a female relative in your family discovers a breast lump how promptly will you seek help?   1. Within one week 2. Within 1 month 3. Within 1-3 months 4. Not bother at all 5. Depends on various factors – Specify __________________________________ | | | | | | | | | | | | | | | | | | | | | | | |  |
| 5.9 | | If you knew that breast cancer can be detected at an early stage would you encourage your wife or female relatives to get screened   1. Yes ***skip to 5.11*** 2. No 3. Not sure | | | | | | |  | | | | | | | 5.10 | | | If No why not?  Please specify  99= Refused to answer | | | | | | |  |
| 5.11 | | If Yes, who would be expected to pay for the screening tests? | | | | | | | | | | | | | | | | | | | | | | | | |
|  |  | 1. Myself 2. Her family 3. My parents | | | | | | | | | | | | | | 1. The Government 2. Depends – Explain 3. Don’t know   99=Declined to answer | | | | | | | | | |  |
| 5.12 | | If your wife was diagnosed with breast cancer whom would you tell? No one   1. Only immediate family 2. CommunityElders 3. Religious leaders 4. Other –Specify/ --------------------------------------------------   ***If answer is 2-6 skip to 5.14*** | | | | | | |  | | | | | | | 5.13 | | | If No one Why not***/***   1. Fear of being ostracized 2. Shame/Embarrassment 3. Taboo 4. Other specify   --------------------------------------------- | | | | | | |  |
| 5.14 | | Whose responsibility would it be to look after/support your wife if she was diagnosed with breast cancer   1. Myself 2. Wife’s family 3. Community 4. Our children 5. Government 6. Other -specify   -------------------------------------------------- | | | | | | |  | | | | | | | 5.15 | | | What would you do if your wife has been living with breast cancer?   1. Support her 2. Leave her 3. Take her back to her parent’s 4. Take another wife 5. I don’t know 6. 99.Refused to answer 7. Other – specify *_________________________* | | | | | | |  |
| Enumerators Code  _____­_______________________________________ | | | | | | | | | | | | | | | | | Enumerators Signature and Date  ___________________________ ____________ | | | | | | | | | |
| Supervisors name  ____________________________________________ | | | | | | | | | | | | | | | | | Supervisors Signature and Date  _______________________ _________________ | | | | | | | | | |
| End time **:_______________** | | | | | | | | | | | | | | | | | | | | | | | | | | |
