# Supplementary material for: Breast Cancer knowledge, perceptions and practices in a rural Community in Coastal Kenya
Source: BMC Public Health. 2019 Feb 12;19:180. doi: 10.1186/s12889-019-6464-3 (PMC6373063; doi:10.1186/s12889-019-6464-3)
Supplement: Supplementary file 2 — Female questionnaire (DOCX 35 kb) [file 12889_2019_6464_MOESM2_ESM.docx]

| \|  \|  \| \| --- \| --- \| \| Name and Signature of Person conducting informed consent \| ***Date*** \| \|  \|  \| | | | | | | | | | | | | | | | | | | | | | | | | | | |  |
| --- | --- | --- | --- | --- | --- | --- | --- | --- | --- | --- | --- | --- | --- | --- | --- | --- | --- | --- | --- | --- | --- | --- | --- | --- | --- | --- | --- | --- | --- | --- | --- | --- | --- |
| **Aga Khan University Hospital, Nairobi**  **Breast Cancer KAP Questionnaire** | | | | | | | | | | | | | | | | | | | | | | | | | | |  |
| Start time | | | |  |  |  |  |  | **Questionnaire code-:** | | | | | | | | | | | |  |  |  | |  | |  |
|  | | | | | | | | | | | | | | | | | | | | | | | | | | |  |
| **CLUSTER NO.** | | | |  |  |  |  |  | Household No | | | | | | | | | | | |  |  |  | |  | |  |
| Village: | | | | | | | | | | | | | | | | | | | | | | | | | | |  |
| Name of household head | | | | | | | | | | | | | | | | | | | | | | | | | | |  |
| Relationship to Respondent  1=Husband***;*** 2=Brother***;*** 3=Father***;*** 4=Uncle***;*** 5=Grandfather***;*** 6=Other (specify) ***________________*** | | | | | | | | | | | | | | | | | | | | | | | |  | | |  |
| **Introduction** | | | | | | | | | | | | | | | | | | | | | | | | | | |  |
| Researchers at The Aga Khan University Hospital, Nairobi are interested in learning how we can improve women’s health in Kaloleni sub-county, Kilifi County particularly as it relates to breast cancer. Breast Cancer involves a growth in the breast that has the ability to spread outside the breast to other parts of the body, and is a public health problem that afflicts women all around the world. In Kenya, breast cancer is the second most common cancer in women after cancer of the cervix. We are interviewing 450 women, ages 15 and above and 250 men from randomly selected households within Kaloleni, to gain a greater insight as to women’s current breast cancer knowledge, attitudes, and beliefs. The collective outcomes of our interview will be used to inform policy on promotion of early detection and timely treatment of breast cancer and establish an appropriate population based breast cancer screening and awareness program Your contribution will be important to conduct this study and your participation will be kept confidential. Before we begin, can you confirm your eligibility to take part in the interview by answering three questions:  Name :_________________________________**DoB: DD/MM/YY___________________________**  Mobile number: **________________________** | | | | | | | | | | | | | | | | | | | | | | | | | | |  |
| 1.0 Questions on Socio demographic Factors | | | | | | | | | | | | | | | | | | | | | | | | | | |  |
| **No.** | | **Question** | | | | | | | | **Code** | | | | | | | **No.** | **Question** | | | | | | | | **Code** |  |
| 1.1 | | Total Number of Household Members living together | | | | | | | |  | | | | | | | 1.2 | Marital status   1. Single 2. Married 3. Divorced 4. Widowed 5. Other : specify | | | | | | | |  |  |
|  |  |  |  |  |  |  |  |  |  |  | | | | | | |  |  |  |  |  |  |  |  |  |  |  |
| 1.3 | | Highest level of Education   1. None 2. Primary 3. Secondary 4. Tertiary 5. Education classes for Mature students 6. Declined to answer | | | | | | | |  | | | | | | | 1.4 | Religion   1. Catholic 2. Protestant 3. Islam 4. Traditional 5. Other   ------------------------------------   1. None 2. Declined to answer | | | | | | | |  |  |
| 1.5 | | Occupation   1. Clerical/Management/Admin 2. Crafts 3. Trader 4. Professional technical 5. Farmer 6. Housewife 7. Other – specify 8. None | | | | | | | | | | | | | | | | | | | | | | | |  |  |
| 2.0 Questions Addressing General Knowledge on Breast Cancer | | | | | | | | | | | | | | | | | | | | | | | | | | |  |
| 2.1 | | Have you heard the word/term Cancer before this survey?   1. Yes 2. No | | | | | | | | |  | | | 2.2 | | | | Have you heard the word/term breast cancer before this survey   1. Yes 2. No | | | | | | | |  |  |
|  |  |  |  |  |  |  |  |  |  |  |  | | |  |  |  |  |  |  |  |  |  |  |  |  |  |  |
| 2.3 | | Have you ever known a person suffering from breast cancer   1. Yes 2. No ***-If no skip to 2.5*** | | | | | | | | |  | | | 2.4 | | | | If Yes who?   1. Friend 2. Mother 3. Sister 4. Daughter 5. Other   ---------------------------------------------- | | | | | | | |  |  |
|  |  |  |  |  |  |  |  |  |  |  |  | | |  |  |  |  |  |  |  |  |  |  |  |  |  |  |
|  | |  | | | | | | | | |  | | |  | | | |  | | | | | | | |  |  |
| 2.5 | | How much do you know about breast cancer     1. Nothing at all 2. I know a little about the disease 3. I am very familiar with it 4. I have only heard the term before 5. Declined to answer | | | | | | | | |  | | | 2.6 | | | | What do you know about Breast cancer? Is it a serious illness?   1. Yes 2. No 3. Don’t know     *(****only answer this if 2.5 is answered as option 2- 4)*** | | | | | | | |  |  |
|  |  |  |  |  |  |  |  |  |  |  |  | | |  |  |  |  |  |  |  |  |  |  |  |  |  |  |
| 2.7 | | Do you know what the cause of breast cancer is? [more than one answer can be selected]   1. A Virus 2. Close contact with person with breast cancer 3. Hereditary (passed from parent to child) 4. Lifestyle choices (foods, smoking, lack of exercise, etc.) 5. Evil eye 6. Witchcraft 7. No-one knows the cause/ 8. I don’t know 9. Declined to answer 10. Other - specify   -------------------------------------------- | | | | | | | | |  | | | 2.8 | | | | Do men get breast cancer   1. Yes 2. No 3. Don’t know | | | | | | | |  |  |
|  |  |  |  |  |  |  |  |  |  |  |  |  |  |  |  |  |  |  |  |  |  |  |  |  |  |  |  |
| 2.9 | | Do you think breast cancer is a curable disease?   1. Yes 2. No 3. Don’t know | | | | | | | | |  | | | 2.10 | | | | Do you think it is possible to survive breast cancer if detected early?     1. Yes 2. No 3. Don’t know | | | | | | | |  |  |
|  |  |  |  |  |  |  |  |  |  |  |  | | |  |  |  |  |  |  |  |  |  |  |  |  |  |  |
|  | |  | | | | | | | | |  | | |  | | | |  | | | | | | | |  |  |
| 2.11 | | Do you think breast disease can be treated by traditional healer?   1. Yes 2. No 3. Don’t know | | | | | | | | |  | | | 2.12 | | | | Do you know the signs and symptoms of breast cancer?     1. Yes 2. No **[Skip to 2.14]** | | | | | | | |  |  |
|  |  |  |  |  |  |  |  |  |  |  |  | | |  |  |  |  |  |  |  |  |  |  |  |  |  |  |
| 2.13 | | If Yes in **Q2.12**, please list at least three signs and symptoms  **--------------------------------------------------------------------------------------------------------------------------**  **-------------------------------------------------------------** | | | | | | | | |  | | | 2.14 | | | | Do you know how breast cancer is diagnosed?     1. Yes 2. No **[Skip to 3.1]** | | | | | | | |  |  |
|  |  |  |  |  |  |  |  |  |  |  |  |  |  |  |  |  |  |  |  |  |  |  |  |  |  |  |  |
|  |  |  |  |  |  |  |  |  |  |  |  |  |  | 2.15 | | | | **If Yes [select one or more response]**     1. Imaging (X ray of breast) 2. Physical exam by doctor –examination of a doctor 3. Biopsy –(Tissue from breast) 4. Other: specify 5. Don’t know 6. Declined to answer | | | | | | | |  |  |
|  |  |  |  |  |  |  |  |  |  |  |  |  |  |  |  |  |  |  |  |  |  |  |  |  |  |  |  |
| 3.0 Questions on Knowledge of Risk Factors, Symptoms, Screening Methods | | | | | | | | | | | | | | | | | | | | | | | | | | |  |
| 3.1 | | Are the following sign and symptoms of breast cancer? | | | | | | | | | |  | | | 3.1a | | | Lump in breast   1. Yes 2. No 3. I don’t know 4. Declined to answer | | | | | | | |  |  |
|  |  |  |  |  |  |  |  |  |  |  |  |  | | |  |  |  |  |  |  |  |  |  |  |  |  |  |
| 3.1b | | Pain in breast   1. Yes 2. No 3. I don’t know 4. Declined to answer | | | | | | | | | |  | | | 3.1c | | | Nipple discharge   1. Yes 2. No 3. I don’t know 4. Declined to answer | | | | | | | |  |  |
|  |  |  |  |  |  |  |  |  |  |  |  |  | | |  |  |  |  |  |  |  |  |  |  |  |  |  |
| 3.1d | | Painless breast lump   1. Yes 2. No 3. I don’t know 4. Declined to answer | | | | | | | | | | | | | | | | | | | | | | | |  |  |
|  |  |  |  |  |  |  |  |  |  |  |  |  |  |  |  |  |  |  |  |  |  |  |  |  |  |  |  |
| 3.2 | | Do you know the risk factors of breast cancer [Ask against each item] Code response as either**:**     1. Yes 2. No 3. I don’t know 4. Declined to answer   ***If 3.2 is 2,3 or 4 skip to 3.3*** | | | | | | | | | | | | | | | | | | | | | | | | |  |
| 3.2a | | Advanced age   1. Yes 2. No 3. I don’t know 4. Declined to answer | | | | | | | | |  | | | | 3.2i | | | | | Early menarche   1. Yes 2. No 3. I don’t know 4. Declined to answer | | | | | |  |  |
| 3.2b | | Poverty   1. Yes 2. No 3. I don’t know 4. Declined to answer | | | | | | | | |  | | | | 3.2i | | | | | Late menopause   1. Yes 2. No 3. I don’t know 4. Declined to answer | | | | | |  |  |
| 3.2c | | Wearing brassieres   1. Yes 2. No 3. I don’t know 4. Declined to answer | | | | | | | | |  | | | | 3.2k | | | | | Use of oral   1. Yes 2. No 3. I don’t know 4. Declined to answer | | | | | |  |  |
| 3.2d | | First child bearing after 30 years   1. Yes 2. No 3. I don’t know 4. Declined to answer | | | | | | | | |  | | | | 3.2l | | | | | Use of tobacco/ tobacco products   1. Yes 2. No 3. I don’t know 4. Declined to answer | | | | | |  |  |
| 3.2e | | Obesity   1. Yes 2. No 3. I don’t know 4. Declined to answer | | | | | | | | |  | | | | 3.2n | | | | | Use of alcohol/ traditional brew   1. Yes 2. No 3. I don’t know 4. Declined to answer | | | | | |  |  |
| 3.2f | | Contact with breast cancer patient   1. Yes 2. No 3. I don’t know 4. Declined to answer | | | | | | | | |  | | | | 3.2o | | | | | A high fat diet   1. Yes 2. No 3. I don’t know 4. Declined to answer | | | | | |  |  |
| 3.2g | | First relative positive family history   1. Yes 2. No 3. I don’t know 4. Declined to answer | | | | | | | | |  | | | | 3.2p | | | | | Big breasts   1. Yes 2. No 3. I don’t know 4. Declined to answer | | | | | |  |  |
| 3.2h | | Long lactating period   1. Yes 2. No 3. I don’t know 4. Declined to answer | | | | | | | | | | | | | | | | | | | | | | | |  |  |
| 3.3 | | Do you know the Screening Methods for early detection of breast cancer?   1. Yes 2. No **[Skip to 4.1]** | | | | | | | | | | | | | | | | | | | | | | | |  |  |
| 3.3a | | Others - :specify________________________________________________________________________ | | | | | | | | | | | | | | | | | | | | | | | |  |  |
| 3.4 | | Monthly breast self-examination     1. Yes 2. No | | | | | | | | | | | | | | | | | | | | | | | |  |  |
| 3.5 | | Regular clinical breast exam (CBE) once a year   1. Yes 2. No | | | | | | | | | | | | | | | | | | | | | | | |  |  |
| 3.6 | | Mammography/imaging in health center or hospital   1. Yes 2. No | | | | | | | | | | | | | | | | | | | | | | | |  |  |
| 4.0 Questions on Practice and Barriers to Practice | | | | | | | | | | | | | | | | | | | | | | | | | | | |
| Breast self-examination is a method used where women such as yourself can check their own breast cancer without needing to see a doctor. | | | | | | | | | | | | | | | | | | | | | | | | | | | |
| 4.1 | | Do you know how to examine your own breasts for abnormality or signs of breast cancer?     1. Yes 2. No**If 2, 3, 4: Skip to 4.3]** 3. I don’t know **[If 2, 3, 4: Skip to 4.3]** 4. Declined to answer **If 2, 3, 4: Skip to 4.3]** | | | | | | | | | | |  | | | 4.2 | | | | If yes, how often do you practice self- breast examination [select the answer that matches your routine     1. Daily 2. Weekly 3. Monthly after menses 4. Once in a while | | | | | | |  |
|  |  |  |  |  |  |  |  |  |  |  |  |  |  | | |  |  |  |  |  |  |  |  |  |  |  |  |
| 4.3 | | If you do not practice self- breast examination regularly what are the reasons [can select or tick more than one answer]   1. I don’t have a breast problem. 2. I don’t feel comfortable doing self- breast examination 3. I do not know how to perform self- breast examination 4. Unsure about its benefit 5. Refused to answer 6. Other -specify reason   ---------------------------------------------- | | | | | | | | | | |  | | | 4.4 | | | | Have you ever had your breasts examined by any health worker? (Clinical Breast Examination - CBE).   1. Yes **[Skip to 4.6](ruka tha 4.6)** 2. No **[Skip to 5.1](ruka tha 5.1)** 3. Declined to answer | | | | | | |  |
|  |  |  |  |  |  |  |  |  |  |  |  |  |  | | |  |  |  |  |  |  |  |  |  |  |  |  |
|  |  |  |  |  |  |  |  |  |  |  |  |  |  | | |  |  |  |  |  |  |  |  |  |  |  |  |
|  |  |  |  |  |  |  |  |  |  |  |  |  |  | | |  |  |  |  |  |  |  |  |  |  |  |  |
|  |  |  |  |  |  |  |  |  |  |  |  |  |  | | |  |  |  |  |  |  |  |  |  |  |  |  |
|  |  |  |  |  |  |  |  |  |  |  |  |  |  | | |  |  |  |  |  |  |  |  |  |  |  |  |
|  |  |  |  |  |  |  |  |  |  |  |  |  |  | | |  |  |  |  |  |  |  |  |  |  |  |  |
| 4.5 | | If NO what is the reason/s for not having a CBE? (Can tick more than one) | | | | | | | | | | | | | | | | | | | | | | | | |  |
|  |  | 1. Lack of finances 2. No time to see a doctor 3. Fear of outcome 4. No signs/symptoms of a breast problem | | | | | | | | | | | |  | | |  | | | 1. Am not aware of CBE 2. Unsure about the benefit of CBE 3. Other - specify*)*________________________ | | | | | | |  |
|  |  |  |  |  |  |  |  |  |  |  |  |  |  |  | | |  |  |  |  |  |  |  |  |  |  |  |
|  |  |  |  |  |  |  |  |  |  |  |  |  |  |  | | |  |  |  |  |  |  |  |  |  |  |  |
|  |  |  |  |  |  |  |  |  |  |  |  |  |  |  | | |  |  |  |  |  |  |  |  |  |  |  |
| 4.6 | | Have you ever had your breasts examined by a traditional healer     1. Yes 2. No 3. Declined to answer | | | | | | | | | | | |  | | | 4.7 | | | Frequency of examination by health worker or traditional healer   1. Once a year 2. 1-3 times a year 3. Once in a while 4. Other – specify   ------------------------------------------------ | | | | | | |  |
|  |  |  |  |  |  |  |  |  |  |  |  |  |  |  | | |  |  |  |  |  |  |  |  |  |  |  |
| 5.0 Questions Addressing Health Seeking Behaviour and Perceived Barriers | | | | | | | | | | | | | | | | | | | | | | | | | | | |
| 5.1 | | | | Where would you go if you or someone in your family is discovered with a breast swelling **/lump?**   1. Health Facility **[Skip to 5.3]** 2. Faith Healer 3. Traditional Doctor 4. Stay at home 5. Self-medication 6. Other – specify*)* | | | | | | | | | |  | | | 5.2 | | | If you do not choose to go to a health facility what would be the reason/s   1. Shortage of doctors 2. No medicines 3. Rude/uncaring staff 4. Long queues 5. Too expensive 6. It’s God’s will 7. Other - specify   ------------------------------------------------ | | | | | | |  |
|  |  |  |  |  |  |  |  |  |  |  |  |  |  |  | | |  |  |  |  |  |  |  |  |  |  |  |
| 5.3 | | | | If you or someone in your family discovers a breast lump how promptly will you seek help?   1. Within one week 2. Within 1 month 3. Within 1-3 months 4. Not bother at all 5. Depends on various factors – Specify | | | | | | | | | |  | | | 5.4 | | | Will you allow a male doctor to examine your breast?   1. Yes 2. No 3. I don’t know 4. Declined to answer | | | | | | |  |
|  |  |  |  |  |  |  |  |  |  |  |  |  |  |  | | |  |  |  |  |  |  |  |  |  |  |  |
| 5.5 | | | | Will you let a male traditional healer examine your breast     1. Yes 2. No 3. I don’t know 4. Declined to answer | | | | | | | | | |  | | | 5.6 | | | Who makes the decision as to where you will seek assistance in the event of having a breast problem?   1. Myself 2. Husband 3. Father 4. Mother 5. In laws 6. Other – specify | | | | | | |  |
|  |  |  |  |  |  |  |  |  |  |  |  |  |  |  | | |  |  |  |  |  |  |  |  |  |  |  |
| 5.7 | | | | Which health facility would you go to seek assistance in the event of having a breast problem? **(Only answer if question 5.1 is 1)**   1. Dispensary 2. Health centre 3. Sub-county hospital 4. County hospital   Teaching and referral hospital | | | | | | | | | |  | | | 5.7a | | | What would be the reason for the choice above?   1. Vicinity to facility 2. Family decision 3. Finances/Transportation 4. Quality of service 5. Other*)_______________* | | | | | | |  |
| Enumerators Code  ____________­_____________________ | | | | | | | | | | | | | | | | | Enumerators Signature and Date  ___________________________ __________ | | | | | | | | | | |
| Supervisors name  ___________________________________________ | | | | | | | | | | | | | | | | | Supervisors Signature and Date  ___________________________ ____________ | | | | | | | | | | |
| End time: | | | | | | | | | | | | | | | | | | | | | | | | | | | |
