# Supplementary material for: Breast Cancer knowledge, perceptions and practices in a rural Community in Coastal Kenya
Source: BMC Public Health. 2019 Feb 12;19:180. doi: 10.1186/s12889-019-6464-3 (PMC6373063; doi:10.1186/s12889-019-6464-3)
Supplement: Supplementary file 3 — Focus group discussion guide. (DOCX 16 kb) [file 12889_2019_6464_MOESM3_ESM.docx]

| **KALOLENI 2015**  **FOCUS GROUP PROTOCOL AND QUESTIONS** | |
| --- | --- |
| FACILITATOR SPEAKS TO FOCUS GROUP PARTICIPANTS: | |
| Thank you for joining us today. You are participating in a focus group on issues related to the early detection of breast cancer. As you know we are concerned about the high rates of death and illness due to cancer. You have been invited to share your attitudes and beliefs about this topic.  We will record your attitudes and beliefs about a number of issues using a tape recorder. These tapes will be transcribed and used to document concerns expressed during this group session.  Focus groups have been used to learn how various groups feel about important issues in their community. There is no right or wrong answers. We want to learn what you think and feel. You are free to express any concerns that you have.  Your comments are confidential. Your name will not appear in any way in the written document of this group.  We are informal so feel free to get refreshments before and after the session. | |
| **1** | Please read your consent form and sign it. |
|  | 1. Please fill out the demographic form. |
|  | 1. (To facilitator: You may decide to give out incentives at the beginning or end of the focus group session. |

**List of consented participants**

| **No.** | **NAME** | **SIGNATURE** | **DATE** |
| --- | --- | --- | --- |
| 1 |  |  |  |
| 2 |  |  |  |
| 3 |  |  |  |
| 4 |  |  |  |
| 5 |  |  |  |
| 6 |  |  |  |
| 7 |  |  |  |

| **KALOLENI 2015**  **FOCUS GROUP PROTOCOL AND QUESTIONS** |
| --- |
| FACILITATOR SPEAKS TO FOCUS GROUP PARTICIPANTS: |
| 1. Does anyone have any questions before we begin the discussion? |
| 1. Now, let’s begin with introducing yourself by first name only and telling us what comes to mind when we say “women’s health.” |
| 1. Please tell me in your own words what the word “cancer” means to you. |
| 1. What does “breast cancer” mean to you? |
| 1. Medical research indicates that early detection of breast cancer can increase treatment options and greatly reduce death rates for women. What do you think about this statement? |
| 1. When the term or phrase “early detection” is used what comes to your mind? What do you think “early detection” means for women within the community? |
| 1. What do you think would be the barriers for women detecting cancers, e.g. Breast cancer, early so that they could have more treatment options? |
| 1. What tests do you know for detecting breast cancer early? |
| 1. What do you know of Breast self- examination 2. a. How often should women conduct breast self-examination?   b. What do you know of mammogram? |
| 1. Why do you think women in the community rarely get breast cancer screening?***?*** 2. What barriers would keep a woman from getting screened for breast cancer? |
| 1. What could health workers in the health field do to make it more comfortable for women to get screenings? |
| 1. Sometimes women will be detected early for breast cancer and then fail to follow-up with the prescribed treatment. 2. Do you know of cases where this has happened? 3. Think about this for breast cancer 4. Think about this for any other cancer/ |
| 1. Why do you think that this happens? 2. Think about this for breast cancer 3. Think about this for any other cancer |
| 1. What was the outcome? 2. Think about this for breast cancer 3. Think about this for any other cancer |
| 1. What do you think is necessary to improve the chances that a woman will follow-up with prescribed treatments? 2. For breast cancer   b. For any other cancer |
| 1. Are there people in the family and community that women are likely to listen to when it comes to talking about early detection of cancer and follow-up treatments for cancer? Who are these people likely to be? |
| 1. Are women likely to go to these people for advice about cancer? What sort of advice or services are these individuals likely to provide? |
| 1. Is there anything else anyone would like to add? |
| Thank you for your time and help with this project. If anyone would like to speak with us afterwards, feel free to do so.  ***Adopted from Racial and Ethnic Approaches to Community Health (REACH) 2010 Program - Division of Preventive Medicine, Department of Medicine, University of Alabama at Birmingham Alabama*** |
