# Supplementary material for: Breast Cancer knowledge, perceptions and practices in a rural Community in Coastal Kenya
Source: BMC Public Health. 2019 Feb 12;19:180. doi: 10.1186/s12889-019-6464-3 (PMC6373063; doi:10.1186/s12889-019-6464-3)
Supplement: Supplementary file 4 — Key Informant interview guide (DOCX 30 kb) [file 12889_2019_6464_MOESM4_ESM.docx]

Thank you for your time to meet with us today. You are participating in a Key informant Interview to discuss issues related to the early detection of breast cancer. As you may be aware we are concerned about the high rates of death and illness due to cancer. As a key stakeholder in the provision of health care services to the community in Kaloleni Sub County, you have been selected to share your attitudes and beliefs about this topic.

We will record your attitudes and beliefs about a number of issues using a tape recorder. These tapes will be transcribed and used to document your opinions and knowledge and concerns about BC

There is no right or wrong answer. We want to learn what you think and feel. You are free to express any concerns that you have.

Your comments are confidential. Your name will not appear in any way in the written document

Please read your consent form and sign it.

Please fill out the demographic form.

Do you have any questions before we begin the discussion?

Now, let’s begin with introducing yourself by first name only and telling us what comes to mind when we say “women’s health.”

| **TARGET GROUP**: **(Doctor/Clinical Officer/Nurse/Community Health Worker)** | |
| --- | --- |
| 1 | What health and social issues are people in Kaloleni Sub County most concerned about? |
| 2 | Are there geographical areas within Kaloleni Sub-County where women are not getting the health services they need? |
| 3 | Do women in the community trust the healthcare system? Why or why not? |
| 4 | Are there barriers (e.g. language, modesty, religion or beliefs, transportation, childcare) that keep women from seeking care? |
| 5 | In your opinion, do you think that cancer is a problem in Kaloleni Sub County? If yes, what are the most common cancers you see in men? And in women in Kaloleni county? |
| 6 | For all cancers- at what stage of disease do patients usually present to a health facility? What about for Breast cancer in women? |
| 7 | Would you please tell us about your experience (e.g. yourself, family members, and friends) with breast cancer? |
| 8 | What do you think causes breast cancer? |
| 9 | How do you feel about preventive care and behaviors for BC, such as maintaining a healthy body weight and reducing alcohol consumption? |
| 10 | What is being done in your community to educate women about breast cancer? Is it working? |
| 11 | What can be done differently in your community to make sure breast health messages and services get to women that need them? |
| 12 | Where would women go to receive clinical breast exam and screening mammography? |
| 13 | What do you think are the barriers that prevent *women in your community* from getting breast cancer screenings every year? |
| 14 | Do you know about breast cancer screening services? (Probe to find out what these are if the answer is “Yes”)   - If the answer is yes: Do you relay this information to your patients? How do you relay this information and what forum/s do you use? What have you found to be the most effective medium/tool to relay this message to the community/patients? - If the answer is no: Would you like to know what type of BC screening services are available and where these services can be accessed. In your experience and opinion, what is the most effective way to educate women in your area about breast health issues? |
| 15 | What breast health education and outreach services does your office/clinic/hospital provide? |
| 16 | How does your office/clinic/hospital reach out to women who do not access breast cancer screening services? |
| 17 | What type of treatment services for Cancers and for BC, does your health facility provide? |
| **Probing questions** | |
| 18 | What are your breast health screening recommendations for women of average risk for breast cancer? |
| 19 | Do you have a method of tracking whether or not women are following your breast health screening recommendations? Please describe your methods. |
| 20 | How do you follow-up with clients who do not show up for appointments? |
| 21 | When women come to you for a checkup for other illnesses at your office/clinic/hospital, do they get a clinical breast exam? |
| 22 | What are the usual number of days between breast cancer diagnosis and the start of treatment? |
| 23 | What gaps and barriers negatively impact a woman’s *transition* from diagnosis to treatment in Kaloleni sub- County? |
| 24 | What are the barriers and challenges experienced by your clients *during* treatment? |
| 25 | **Closing Question: (To all)**  What new services, programs, and policies are needed in your area to deliver effective breast health services? |
| ***Adopted from*** ***Central Texas Affiliate of Susan G. Komen®*** (Qualitative Data: Ensuring Community Input) | |
